# Partial Discharge Ultrasound Detection Using the Sagnac Interferometer System

**DOI:** 10.3390/s18051425

**Published:** 2018-05-04

**Authors:** Yu Wang, Xiaomin Li, Yan Gao, Hongjuan Zhang, Dong Wang, Baoquan Jin

**Affiliations:** 1 Key Laboratory of Advanced Transducers and Intelligent Control Systems (Ministry of Education and Shanxi Province), College of Physics and Optoelectronics, Taiyuan University of Technology, Taiyuan 030024, China; wangyu@tyut.edu.cn (Y.W.); wangdong@tyut.edu.cn (D.W.); 2 College of Electrical and Power Engineering, Taiyuan University of Technology, Taiyuan 030024, China; lixiaomin0157@link.tyut.edu.cn (X.L.); gaoyan@tyut.edu.cn (Y.G.); zhanghongjuan@tyut.edu.cn (H.Z.); 3 State Key Laboratory of Coal and CBM Co-mining, Jincheng, Shanxi 048000, China

**Keywords:** partial discharges, optical fiber sensor, ultrasound detection, time-frequency analysis

## Abstract

Partial discharge detection is crucial for electrical cable safety evaluation. The ultrasonic signals frequently generated in the partial discharge process contains important characteristic information. However, traditional ultrasonic transducers are easily subject to strong electromagnetic interference in environments with high voltages and strong magnetic fields. In order to overcome this problem, an optical fiber Sagnac interferometer system is proposed for partial discharge ultrasound detection. Optical fiber sensing and time-frequency analysis of the ultrasonic signals excited by the piezoelectric ultrasonic transducer is realized for the first time. The effective frequency band of the Sagnac interferometer system was up to 175 kHz with the help of a designed 10 kV partial discharge simulator device. Using the cumulative histogram method, the characteristic ultrasonic frequency band of the partial discharges was between 28.9 kHz and 57.6 kHz for this optical fiber partial discharge detection system. This new ultrasound sensor can be used as an ideal ultrasonic source for the intrinsically safe detection of partial discharges in an explosive environment.

## 1. Introduction

The online monitoring and positioning of partial discharges (PD) is very important for the state maintenance of power appliances. The PD process is always accompanied by ionization, thermal radiation, acoustic emission, photon emission and other physical phenomena [1], and it also includes a chemical reaction resulting in SF_6_ and other gases [2]. Based on these physical phenomena, a variety of PD detection methods are gradually derived, which can specifically be divided into electrical and non-electrical detection methods.

The electrical detection methods include the pulse current method, ultra-high frequency detection method, dielectric loss analysis method, and radio interference voltage method [3,4,5,6,7], which are susceptible to electromagnetic interference and may be the cause of pollution to the power grid and power equipment. Therefore, non-electrical measurement methods are gradually attracting more attention, because of their strong ability of anti-electromagnetic interference, including the ultrasonic method, chemical method, infrared thermal detection method and fiber-optic method [8,9,10,11].

It is well known that PD pulses generally emit weak ultrasonic signals from 20 kHz to 300 kHz. The huge electromagnetic interference generated by power appliances often limits the traditional piezoelectric transducer (PZT)-type of acoustic emission sensor from being widely used. On the other hand, as an alternative, optical fiber interferometer-based acoustic sensors that are immune to electromagnetic interferences [12,13] can be employed within an environment, such as inside or outside power transformers for PD monitoring, as well as source locating [14].

There are several major types of fiber-optic acoustic sensors that have been developed recently, including the diaphragm-based Fabry–Perot interferometric (FPI) sensors [15,16,17], fiber Bragg grating (FBG)-based sensors [18,19], Sagnac interferometric sensors [15,20,21,22], and fiber laser-based interferometric sensors [23,24]. These types of sensors, depending on their structure, have different sensing performances in terms of the sensitivity and frequency response, and have different applicability, such as being capable of being placed inside or outside transformers, or both [20,25,26]. The sensitivity of the FPI is determined by the structure and material of the interferometer cavity [27]. Their low reflectance and multilayer structure result in a low signal-to-noise ratio (SNR) and the manufacturing process is complex [28]. It can only achieve point-based measurement. The technology of the FBG method requires multiple FBG multiplexing to measure the full range of partial discharge [29], showing a strong dependence on the technology of integration, and the stability needs to be strengthened [30].

The Sagnac interferometry principle and demodulation method are simple and can realize distributed measurement. As a counter case, the Sagnac interferometric sensor shows its superior stability against the influence of environmental temperature, due to its truly path-matched interference mode. Besides this, the Sagnac interferometric sensor also has high sensitivity and an excellent high-frequency response to ultrasonic signals, which render it particularly suitable for PD acoustic sensing. A push–pull acoustic fiber-optic sensor from a push–pull acoustic transducer incorporated in a Sagnac interferometer was used to detect ultrasonic signals in the frequency range 0.4–1.5 MHz [31]. A laser ultrasonic system containing an array of fiberized laser ultrasonic sources was detected by the embedded optical fiber Sagnac ultrasonic sensor [32]. The Sagnac interferometer fiber optic sensor is suitable for the detection of small displacements with high frequency, such as the ultrasonic waves used in conventional nondestructive testing [33]. However, there are relatively few studies on the standard ultrasonic signals detected by the optical fiber method and detailed analysis from the experimental point of view. Therefore, it is necessary to study in depth the time-frequency characteristics of PD signals with the purpose of realizing more efficient and accurate identification by means of the fiber-optic method.

In this study, we carried out an experimental investigation into PD detection using the fiber-optic method. The time-frequency characteristics of PD signals detected by the linear Sagnac system were firstly analyzed and compared with the experimental results of the traditional piezoelectric transducer. The influence of various sensing probes on the characteristics of PD detection were innovatively studied. Compared with other point-like ultrasonic sensors such as FBG [34,35], the first advantage of this proposed optical fiber sensing probe consisting of a fiber coil is the ability of long distance detection while the FBG sensor is limited by the optical power attenuation existing in the transmission gratings, whose function is similar to a bandpass filter [36,37]. Secondly, the fiber coil is easy to implement and can be modified according to different field environments, which makes it suitable for field tests and applications. In contrast, the FBG needs to be prepared and encapsulated in advance [38,39], and thus lacks flexibility. Thirdly, the multi-sensor fusion scheme is an important solution for ultrasonic detection [40]. Hence, different fiber coils could be simultaneously used for the detection of components with different characteristic frequencies of PD ultrasonic signals in order to enhance the detection performance of PD signals.

## 2. Detection of Ultrasonic Signals by Interferometric Sagnac

In the operation of power equipment, there is a higher electric field intensity than the surrounding area, which leads to the phenomenon of electric field agglomeration. The PD process may lead to the elevation of local temperature, the deterioration of insulation, and the breakdown of insulation material, which then generates a leakage current. PD produces ultrasonic waves and electromagnetic waves with the increase of PD intensity, thereby causing a short circuit fault of electrical equipment.

### 2.1. Mechanism of Partial Discharges That Produce Ultrasonic Signals

**Figure 1:**
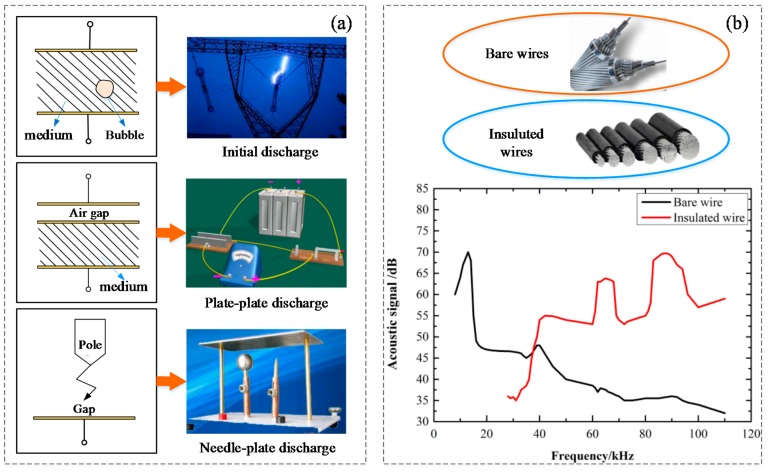
(**a**) The common types of partial discharges (PD); (**b**) bare wire and insulated wire and the discharge spectrum of bare wire and insulated wire in the air.

In the running of electrical equipment, vibration, thermal expansion, cracking, and material detachment phenomena may occur; electric insulating materials produce the ionization of charged particles. The physical properties of charged particles are worse under the repeated impact of the charged particles in the air gap surface. The insulating materials then lose their original performance, which leads to a chemical reaction, as well as generating SF_6_ and causing a partial discharge. The common types of PD are shown in Figure 1a.

The two-order equation of the elastic force of the bubble follows the following equation [41]:

(1) $$L_{m}C_{m}\frac{d^{2}u_{c}}{dt^{2}}+R_{m}C_{m}\frac{du_{c}}{dt}+u_{c}=0$$

where *L_m_* is the inductance of the mechanical equivalent circuit; *C_m_* is a parameter describing the physical structure of the system, which represents the elasticity of a system; *R_m_* is a parameter describing the physical structure of a system, which represents a system with frictional losses; and *u_c_* is the external force of the bubble wall.

In general, in the oil medium, where the resistance is small, the equation is:

(2) $$R<2\sqrt{\frac{L}{C}}$$

It has been shown that the mechanical process of PD in an air bubble is an oscillatory process.

*u_c_* is multiplied by the surface area of the bubble, that is, the acoustic pressure of the ultrasound, ignoring the oscillation of PD and the volume change of the bubble, then *u_c_* is proportional to the sound pressure of the ultrasonic wave [42]:

(3) $$u_{c}=\frac{U_{0}\omega_{0}}{\omega }e^{\delta t}\sin(\omega t+\beta )$$

where $\delta =\frac{R_{m}}{2L_{m}}$, $\omega =\sqrt{\frac{1}{L_{m}C_{m}}-{\left(\frac{R_{m}}{2L_{m}}\right)}^{2}}$, $\omega_{0}=\sqrt{\delta^{2}+\omega^{2}}$, $\beta =\mathrm{arct}g\frac{\omega }{\delta }$.

It can be seen that when PD occurs in the bubble, the bubble is attenuated by the impulse electric field force.

(4) $$U_{0}=U_{e}=qE$$

Ignoring the PD oscillation process, from Equations (3) and (4) we can draw the conclusion that the amplitude of the ultrasonic voltage is proportional to the actual discharge.

Under the condition of PD, the amplitude of the ultrasonic signal is proportional to the electric discharge. When the bubble is subjected to PD, the external force *F* is:

(5) $$F=qE=q\frac{U_{c}}{d_{c}}=kq$$

The quality of bubble increases with the increase of the bubble radius. The environment of the bubble is unchanged, the force *F* in the equivalent circuit is unchanged, and the equivalent inductance *L_m_* increases, resulting in the frequency spectrum of the ultrasonic signal moving to a low frequency. With the increase in the discharge, the ultrasonic spectrum moves to a low frequency.

The two main factors that influence the PD ultrasonic wave in the medium are as follows: one is the electric field force in the discharge process; the other is the bubble expansion pressure wave caused by the heating of the discharge arc.

The two basic elements of sound wave propagation are particles and media, and the generation of ultrasonic waves depends on the mass of the high-frequency vibrations and the mass of the elastic medium.

### 2.2. Characteristics of Partial Discharges That Produce Ultrasound

PD produces acoustic pulses with a wide frequency band. Its spectrum and size are not only affected by the test environment and the discharge state and the propagation process, but are also related to the specific type of discharge.

The acoustic spectrum is generated by the wires without an insulating layer and the wires with an insulating layer. Since the frequency of the sound waves is different, their energy is different. Specifically, the frequency of the bare wires is concentrated at 10~50 kHz, as shown in Figure 1b. It has a very fast decay of strength, basically the same as the tip of the flat plate discharge process; the wire insulation layer obtained by the spectrum is approximately equal to the discharge surface insulation [43]. In summary, we can see that the most abundant energy is in the low frequency band of ultrasound.

The PD phenomenon often occurs inside electric power equipment. When the ultrasonic wave is received, the sensor is usually placed on the shell of the device. Therefore, some attenuation occurs in the process of reaching the detection point from the source of the sound wave and the different propagation medium. The propagation speed of the sound waves is also different. PD ultrasonic waves can be seen as a point source and ultrasonic waves in the form of spherical waves spread to the surrounding area. In the process of ultrasonic propagation, the energy of the sound waves decreases as the propagation distance increases, which is called attenuation. The acoustic attenuation is related to the acoustic frequency, and the higher the frequency, the greater the attenuation. Therefore, the increase in the sensor after the preamplifier must be high enough for detection.

The propagation of ultrasound in the medium will also have a strong direction, thus the process of propagation will be reflected in the interface of the media. The occurrence of a reflection will reduce the sound energy during the propagation [43]. Ultrasonic radiation is at a critical angle of 26° in the air, and if the amplitude of the incident angle exceeds the value of the ultrasonic wave, it will produce total reflection. Thus, the angle of the ultrasonic radiation and the installation of the sensor probe should be highly valued in the actual test.

The characteristics of ultrasonic waves related to discharge includes signal amplitude, signal frequency, and signal duration. With the increase of discharge, the ultrasonic spectrum moves to a low frequency. In the needle plate PD model, the relationship between the three characteristic quantities and the discharge volume *q* is as follows [44]

(6) $$\left\{ \begin{array}{l} A=1.128\times 10_{}{}^{-5}q-0.323\\ F=-2.027\times 10_{}{}^{-5}q+89.91\\ T=5.231\times 10_{}{}^{-5}q+75.49 \end{array}\right.$$

where *A* represents the magnitude of the ultrasonic amplitude, *q* represents the discharge quantity, *F* represents the ultrasonic frequency, and *T* represents the signal duration time [35].

### 2.3. Partial Discharge Generating Device

**Figure 2:**
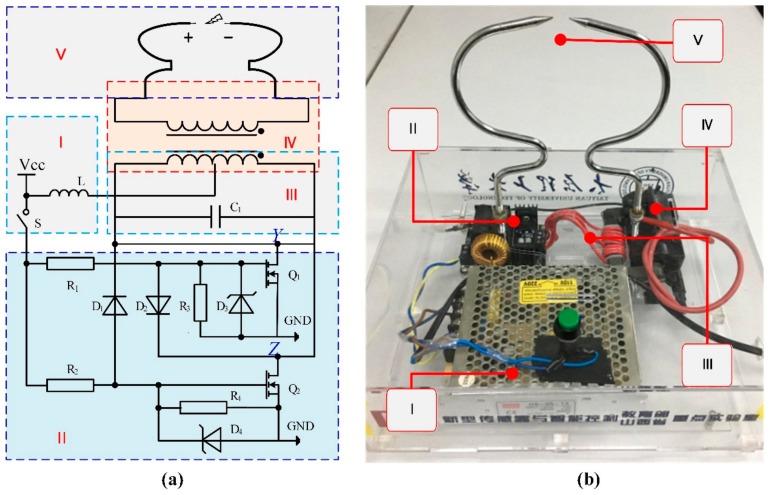
PD simulator device: (**a**) circuit diagram and (**b**) physical diagram in the laboratory. Part I: power supply module; part II: DC–AC conversion circuit; part III: *LC* oscillation circuit; part IV: high voltage coil; part V: needle-needle electrode.

Based on the above theory, we built a PD simulator device, which was used to generate PD signals. Figure 2 shows the PD simulator device, which was composed of five parts: the power supply module, the DC–AC conversion circuit, the *LC* oscillation circuit, the high voltage coil and the needle–needle electrode.

Part I provided power to the simulator device. The port V_CC_ provided a DC voltage of 12 V and a current of 10 A. The inductance *L* was the smoothing reactor, which was used to prevent the circuit from being destroyed by pulling a huge peak current from the power. Part II converted the DC voltage into AC voltage by using the thyristor breaker in the push–pull circuit. Part III played the role of filter and controlled the on–off of the thyristor Q_1_ and thyristor Q_2_. It also prevented the thyristor Q_1_ and thyristor Q_2_ from exploding. The ratio of part IV was 1:800, through which the voltage is increased to 10 kV. When the switch S was closed and the circuit was powered, the positive terminal voltage of needle–needle electrode was 10 kV, but the negative terminal voltage was 0 V. Then, the high voltage broke through the air and PD was generated between the needles. The physical PD simulator device was based on the principle of discharge is shown in Figure 2b. In this paper, we used the optical fiber PD detection system to carry out the experiment with the PD simulator device.

## 3. Optical Fiber Sensing System

**Figure 3:**
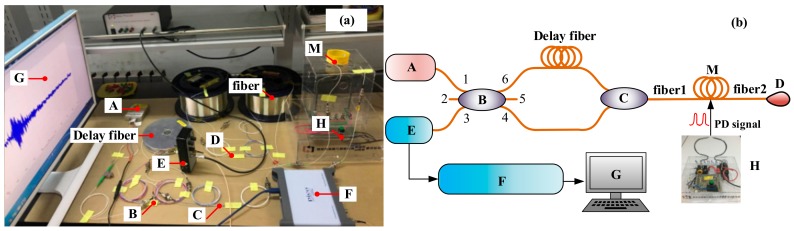
Optical fiber PD detection system (OFPDS). (**a**) Experimental device diagram and (**b**) the experimental schematic diagram. A—Laser, B—3 × 3 Coupler, C—2 × 1 Coupler, D—1 × 2 Coupler, E—Detector, F—Data acquisition (DAQ) card, G—Personal computer (PC), H—PD simulator device, M—optical fiber sensor.

The principle of the optical fiber PD detection system (OFPDS) is shown in Figure 3. A was the amplified spontaneous emission (ASE) source, B was the 3 × 3 coupler, C was the 2 × 1 coupler, D was the 1 × 2 coupler, E was the balanced detector, F was the Data acquisition (DAQ) card, G was the host computer, and H was the PD simulator device shown in Figure 2. M was the optical fiber sensing probe.

The laser beam was emitted by the light source to form four light path loops by the coupler B. It can be listed as follows: Path ① port 6- delay fiber- C- sensing fiber 1- M- sensing fiber 2- D- sensing fiber 2- M- sensing fiber 1- C- delayed fiber- port 6. Path ② port 6- delay fiber- C- sensing fiber 1- M- sensing fiber 2- D- sensing fiber 2- M- sensing fiber 1- C- port 4. Path ③ port 4- C- sensing fiber 1- M- sensing fiber 2- D- sensing fiber 2- M- sensing fiber 1- C- port 4. Path ④ port 4- C- sensing fiber 1- M- sensing fiber 2- D- sensing fiber 2- M- sensing fiber 1- C- delay fiber- port 6.

In the optical path ② and the optical path ④, the time of the laser at which the PD signals were generated by the PD simulator device H was different due to the different time through the delay fiber. The interference then occurred in the coupler B and the change of the phase was converted into light intensity variety. The interference of optical signals was converted into electrical signals in detector E. The data collection and processing were dealt with the DAQ card, finally the host computer displays the output signals.

According to the Sagnac effect, the optical power of the interference signal of the two beams at E is *P*(t):

(7) $$\begin{array}{l} P(t)=\frac{P_{0}}{3}\left\{1+\cos\left[\Delta \Psi +\varphi \left(t-\tau_{1}\right)+\varphi \left(t-\tau_{2}\right)-\varphi \left(t-\tau_{3}\right)-\varphi \left(t-\tau_{4}\right)\right]\right\}\\ \begin{array}{cc} & =I^{dc}\left(t\right)+I^{ac}\left(t\right) \end{array} \end{array}$$

In the above equation, *P*_0_ is the input optical power, *φ*(*t*) is the vibration caused by the phase shift; Δ*Ψ* is the signal caused by the constant non-reciprocal phase shift. *τ*_1_ and *τ*_2_ are respectively the delay time of the clockwise light that twice goes through the vibration point where the PD signals act. *τ*_3_, *τ*_4_ are respectively the delay time at which the counter-clockwise light twice goes through the vibration source. The term *I^dc^*(*t*) presents the direct current (DC) component and the term *I^ac^*(*t*) presents the alternating current (AC) component of the optical power.

The ultrasonic signal could be expressed as a set of sinusoidal waves with different amplitudes, frequencies, and initial phases, shown as follows:

(8) $$y(t)=\sum_{i=1}^{N}\varphi_{0}\sin\left(\omega_{i}t+\varphi_{i}\right)$$

where *y*(*t*) is the change of optical phase as the variation of time, *φ*_0_ is the amplitude of the *i*-th sinusoidal wave, *ω_i_* is the angular frequency of the *i*-th sinusoidal wave, *φ_i_* is the initial phase of the *i*-th sinusoidal wave, and *N* is the integer number of the sinusoidal wave.

Assume that the length of delay fiber in the system is *L*_d_, the total length of sensing fiber is *L*, the distance between the sensing probe position *M* and the output of the coupler *C* is *L*_1_, the distance between the sensing probe position *M* and the output of the coupler *D* is *L*_2_, the velocity of light propagating in a vacuum is *c*, and the refractive index of the optical fiber is *n*. In this case, the different delay times in Equation (7) could be expressed as follows:

(9) $$\begin{array}{l} L=L_{1}+L_{2}\\ \tau_{1}=\frac{\left(L_{d}+L_{1}\right)n}{c},\tau_{2}=\frac{\left(2L_{2}+L_{1}\right)n}{c}\\ \tau_{3}=\frac{L_{1}n}{c},\tau_{4}=\frac{\left(2L_{2}+L_{1}+L_{d}\right)n}{c} \end{array}$$

The direct current (DC) component and the alternating current (AC) component *I*^ac^(*t*) of the phase shift could be simplified as follows:

(10) $$\begin{array}{l} I^{dc}\left(t\right)=\frac{P_{0}}{3}\left\{1-\frac{1}{2}\cos\left[\varphi_{0}\sin\left(\omega_{s}t-\tau_{1}\right)+\varphi_{0}\sin\left(\omega_{s}t-\tau_{2}\right)-\varphi_{0}\sin\left(\omega_{s}t-\tau_{3}\right)-\varphi_{0}\sin\left(\omega_{s}t-\tau_{4}\right)\right]\right\}\\ \begin{array}{ccc} & & \approx \frac{P_{0}}{3}\left(1-\frac{1}{2}\right)=\frac{P_{0}}{6} \end{array}\\ I^{ac}\left(t\right)=\frac{P_{0}}{3}\frac{\sqrt{3}}{2}\sin\left[\varphi_{0}\sin\left(\omega_{s}t-\tau_{1}\right)+\varphi_{0}\sin\left(\omega_{s}t-\tau_{2}\right)-\varphi_{0}\sin\left(\omega_{s}t-\tau_{3}\right)-\varphi_{0}\sin\left(\omega_{s}t-\tau_{4}\right)\right]\\ \begin{array}{ccc} & & \approx \end{array}\frac{\sqrt{3}P_{0}}{6}\left[\varphi_{0}\sin\left(\omega_{s}t-\tau_{1}\right)+\varphi_{0}\sin\left(\omega_{s}t-\tau_{2}\right)-\varphi_{0}\sin\left(\omega_{s}t-\tau_{3}\right)-\varphi_{0}\sin\left(\omega_{s}t-\tau_{4}\right)\right] \end{array}$$

where *P*_0_ is the input optical power, and *ω*_s_ is the angular frequency of the ultrasonic wave.

The integral form *I^ac^*(*t*) is also obtained because the phase shift could be considered as the accumulation effect within the length of the optical fiber sensing circles at the position *L*_2_, when *α*_dB_ is the attenuation coefficient of the optical power of the sensing optical fiber, shown as follows:

(11) $$I^{ac}\left(t\right)\approx \int 2\sqrt{3}P_{0}\alpha_{\mathrm{dB}}\varphi_{0}\sin\left(\frac{\omega_{s}nL_{d}}{2c}\right)\cos\left(\omega_{s}\frac{nL_{2}}{c}\right)\cos\left(\omega_{s}t-\frac{\omega_{s}\left(L_{d}+2L\right)n}{c}+\varphi_{i}\right)d_{L_{2}}$$

Assuming that the number of sensing fiber circles is *N*_c_, the strength coefficient of the phase shift of the *j*-th fiber circle is *φ_j_*, the length of the *j*-th fiber circle is *l_j_*, the total alternating current component *I*^ac^(*t*) of the phase shift generated by the ultrasonic wave and detected by all fiber circles could be expressed as follows:

(12) $$I^{ac}\left(t\right)\approx \sum_{j=1}^{N_{c}}\int_{L_{2}}^{L_{2}+l_{j}}2\sqrt{3}P_{0}\alpha_{\mathrm{dB}}\varphi_{j}\sin\left(\frac{\omega_{s}nL_{d}}{2c}\right)\cos\left(\omega_{s}\frac{nL_{2}}{c}\right)\cos\left(\omega_{s}t-\frac{\omega_{s}\left(L_{d}+2L\right)n}{c}+\varphi_{i}\right)d_{L_{2}}$$

Equation (12) could be simplified after the integral operation, shown as follows:

(13) $$\begin{array}{l} I^{ac}\left(t\right)\approx \sum_{j=1}^{N_{c}}\{2\sqrt{3}P_{0}\alpha_{\mathrm{dB}}\varphi_{j}\sin\left(\frac{\omega_{s}nL_{d}}{2c}\right)\cos\left(\omega_{s}t-\frac{\omega_{s}\left(L_{d}+2L\right)n}{c}+\varphi_{i}\right)\\ \frac{c}{\omega_{s}n}\left[\sin\left(\omega_{s}\frac{nL_{2}+nl_{j}}{c}\right)-\sin\left(\omega_{s}\frac{nL_{2}}{c}\right)\right]\} \end{array}$$

As there is always a fiber looseness in the sensing probe, the length of the *j*-th fiber circle *l_j_* could be considered as the circumference of an ellipse. The fiber looseness was determined by the length of the short axis of this ellipse, which was limited to the diameter of the fiber mounts *d_j_*, and also by the inclination angle *θ_j_* during the twining. Thus, the long axis of this ellipse was *d_j_*/cos(*θ_j_*). Therefore, the length of the *j*-th fiber circle *l_j_* could be expressed as follows:

(14) $$l_{j}=\pi d_{j}+2d_{j}\left(\frac{1}{\cos\theta_{j}}-1\right)$$

Finally, the total alternating current component *I*^ac^(*t*) of the phase shift generated by the ultrasonic wave is a function of fiber looseness (*d_j_* and *θ_j_*), sensing distance (*L*_2_), the number of the sensing fiber circles (*N*_c_), and the angular frequency of the ultrasonic wave (*ω_s_*), shown as follows:

(15) $$\begin{array}{l} I^{ac}\left(t\right)\approx \sum_{j=1}^{N_{c}}\{2\sqrt{3}P_{0}\alpha_{\mathrm{dB}}\varphi_{j}\sin\left(\frac{\omega_{s}nL_{d}}{2c}\right)\cos\left(\omega_{s}t-\frac{\omega_{s}\left(L_{d}+2L\right)n}{c}+\varphi_{i}\right)\\ \frac{c}{\omega_{s}n}\left[\sin\left(\omega_{s}\frac{nL_{2}+n\pi d_{j}+2nd_{j}\left(\frac{1}{\cos\theta_{j}}-1\right)}{c}\right)-\sin\left(\omega_{s}\frac{nL_{2}}{c}\right)\right]\} \end{array}$$

As the terms of the difference of the two sine functions in Equation (15) may be positive or negative, the accumulation of phase shift can randomly increase or decrease in the form of a nonlinear and fluctuant variation.

**Figure 4:**
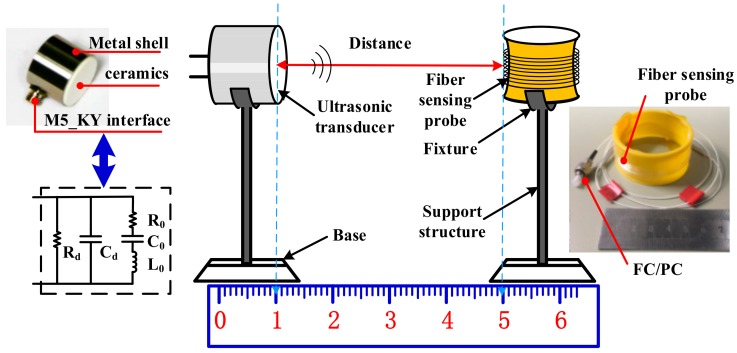
Experimental setup and diagram for the detection of piezoelectric ultrasonic signals.

The optical fiber sensing probe is fused from a 1550 nm single-mode jumper and bare fiber. The diameter of this optical fiber sensing probe was 6 cm, as shown in Figure 4. The length of the optical fiber sensing probe increases with the number of laps. When it increases by one lap, the length of the fiber of the optical fiber sensing probe increases 0.19 m. In this paper, we studied the optimal detection position, turn number, and fiber length of the optical fiber sensing probe, and explored the relationship between the lap number of the optical fiber sensing probe and the PD signal.

## 4. Experiments and Results

### 4.1. Detection of Standard Ultrasonic Signals

OFPDS was used to detect the known standard ultrasonic signal that was generated by the ultrasonic transducer to verify that the OFPDS could detect the standard ultrasonic signal. In this work, we used an air-coupled and non-focus transducer. As shown in Figure 4, the ultrasonic transducer on the left emitted the known standard ultrasonic signal, and the optical fiber sensing probe on the right detected the ultrasonic signals and then converted them so that they carried vibratory position light signals. The central frequency of the ultrasonic transducers were respectively 25 kHz, 58 kHz and 175 kHz. The driving voltage (V_pp_) of the signal generator was set to 20 V and the frequencies of the signal generator were respectively set to 25 kHz, 58 kHz and 175 kHz, in order to generate the mechanical vibration of the elastic thin film in the ultrasonic transducer with the help of the piezoelectric effect.

**Figure 5:**
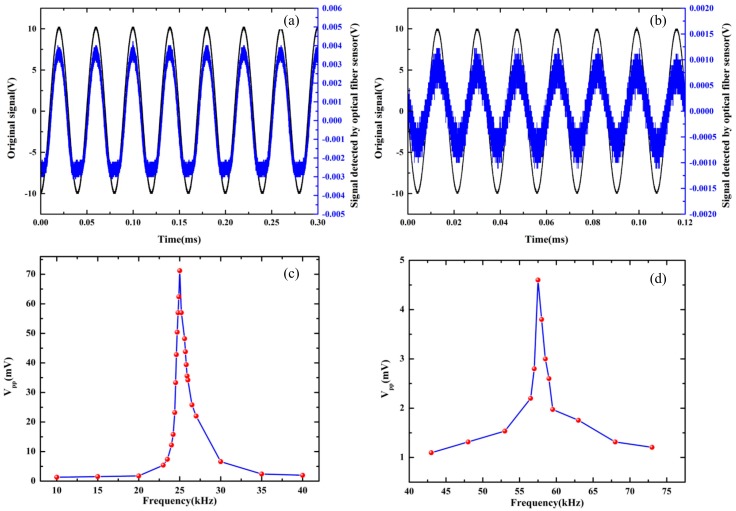
Comparison of original exciting signals (in black) and detected signals (in blue) of the optical fiber sensor at (**a**) 25 kHz and (**b**) 58 kHz. Frequency response characteristics for the ultrasonic transducer of (**c**) 25 kHz and (**d**) 58 kHz.

The sinusoidal ultrasonic signal was detected by the optical fiber sensing probe, and then the signals were detected by the OFPDS, as shown in Figure 5. The distance between the optical fiber sensing probe and the ultrasonic transducer was 4 cm, the V_pp_ of the sinusoidal signal in black from the generator was 20 V, but the sinusoidal ultrasonic signal detected by the optical fiber sensing probe in blue was different. At the same distance, the V_pp_ of the sinusoidal ultrasonic signal detected by the optical fiber sensing probe in blue was 8 mV, the central frequency of the ultrasonic transducer was 25 kHz, as shown in Figure 5a. The V_pp_ was 2 mV with a central frequency of the ultrasonic transducer of 58 kHz, as shown in Figure 5b. The domain signal detected by the OFPDS in blue was consistent with the original signal in black.

As shown in Figure 5a,b, with the same input voltage of 20 V, the V_pp_ amplitudes were different for the 25 kHz and 58 kHz transducers. In fact, because the material, size and capacitance of the two different transducers were completely different, the driving ability and the output power of the ultrasonic transducer were different even with the same input voltage. Meanwhile, for the same transducer, some experiments were carried out in order to investigate the output power of the transducer with the change of excitation frequency. Here, we used a broadband ultrasonic transducer that directly detects the ultrasound generated by the 25 kHz and 58 kHz transducers. With the same input voltage of 20 V, we changed the excitation frequencies in the range of 10 kHz to 40 kHz for the 25 kHz transducer, and in the range of 43 kHz to 73 kHz for the 58 kHz transducer. The detected amplitudes of the sinusoidal signals V_pp_ are plotted in Figure 5c,d. The maximum amplitude at the characteristic frequencies of 25 kHz and 58 kHz can be observed, respectively. These two curves present the frequency response of two transducers.

**Figure 6:**
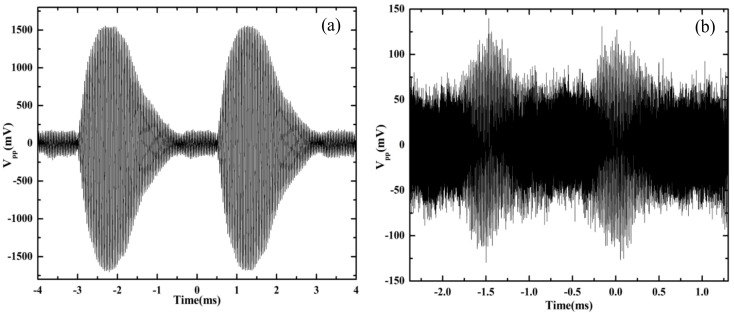
Detection of the tone-burst sinusoidal ultrasonic signal with the transducer of (**a**) 25 kHz and (**b**) 58 kHz.

In contrast to the continuous ultrasonic signal, we also studied the signal changes detected by the optical fiber sensor when the applied signal was a non-continuous ultrasonic signal. The tone-burst sinusoidal ultrasonic signal was detected by the optical fiber sensing probe shown in Figure 6. The excitation signals in the generator area produced a series of 20 sinusoidal waves with an amplitude of 20 V, and the repetition period was 3.5 ms for 25 kHz and 1.5 ms for 58 kHz. The distance between the probe and the ultrasonic transducer was 4 cm. The detected sinusoidal ultrasonic signals *V*_pp_ driven by the 25 kHz and 58 kHz transducers are shown in Figure 6a,b. A better signal-to-noise ratio was obtained for the transducer with a lower frequency.

**Figure 7:**
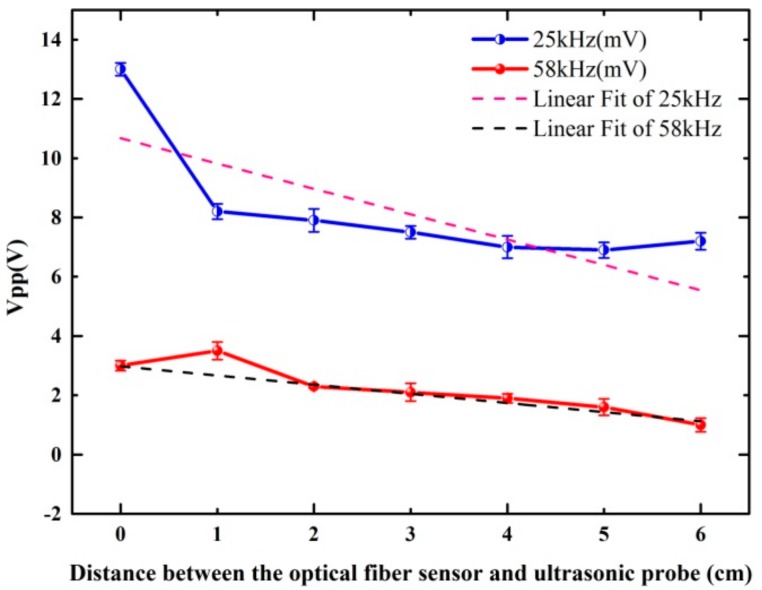
V_pp_ with the variation of distance in the 25 kHz and 58 kHz ultrasonic signals.

In order to further study the influence of different distances on the time-domain signals, further experiments were carried out. The distance between the optical fiber sensing probe and the ultrasonic transducer was set from 0 cm to 6 cm, with 1 cm for each step. Then the signal generator generated a sinusoidal signal, which was applied to the ultrasonic transducer to send out standard ultrasonic signals of 25 kHz and 58 kHz. The ultrasonic signal was then detected by the optical fiber sensing probe, and the V_pp_ with the variation of distance was shown in Figure 7. From the linear fit of the 25 kHz and 58 kHz curves, we can clearly see that as the distance increased, the V_pp_ gradually decreased.

**Figure 8:**
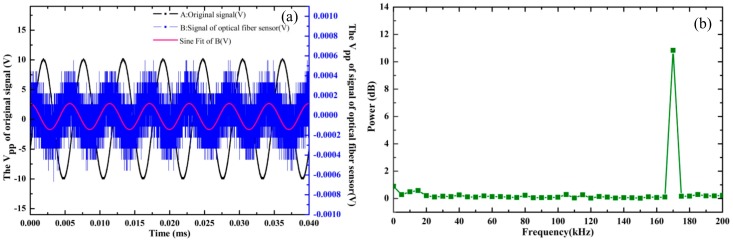
(**a**) The exciting, detected and fitted ultrasonic signals at 175 kHz; (**b**) the frequency spectrum of ultrasonic signals at 175 kHz.

The V_pp_ of the signal generator was set to 20 V and the frequency of the signal generator was set to 175 kHz. Then the signal generator generated a sinusoidal signal in black, which was applied to the ultrasonic transducer to send out a standard ultrasonic signal of 175 kHz. The ultrasonic signal was detected by the optical fiber sensing probe; the signal was not able to be detected when the ultrasonic transducer was 0 cm or further from the fiber sensor. In this case, we wound the fiber around the metal shell of the ultrasonic transducer, with a fiber lap number of 10. The signals in blue detected by the OFPDS in the time-domain are shown in Figure 8a. The V_pp_ of the sine fit of the ultrasonic signals detected by the optical fiber sensing probe in red was 0.2 mV. As the detected signal was weak, the frequency spectrum of the ultrasonic signals was verified using OFPDS. The peak at 175 kHz showed good consistency with the central frequency of the 175 kHz ultrasonic transducer, as shown in Figure 8b.

### 4.2. Detection of Ultrasonic Signals Using PD

**Figure 9:**
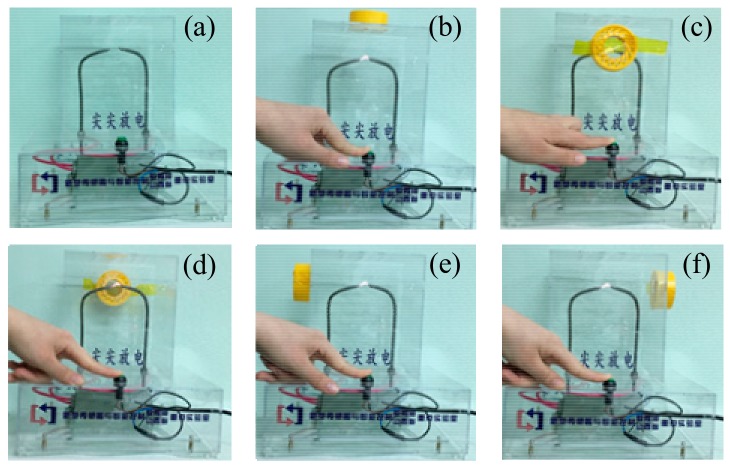
Detection experiments using the optical fiber sensing probe in different positions. (**a**) No PD event. The sensing probe is placed (**b**) on the top, (**c**) in the front, (**d**) behind, (**e**) on the left side and (**f**) on the right side of the discharge protection hood.

The experiment was carried out with the OFPDS platform. In the OFPDS platform, the length of the delay fiber was 2 km, the length of sensing fiber 1 was 4 km and the length of sensing fiber 2 was 4 km. The optical fiber sensing probe shown in Figure 3 was placed 4.1 km away from the start of the OFPDS, and the sensing probe was placed in different positions on the PD model. We made the needle–needle discharge in an orderly manner by triggering the switch button, as shown in Figure 9.

**Figure 10:**
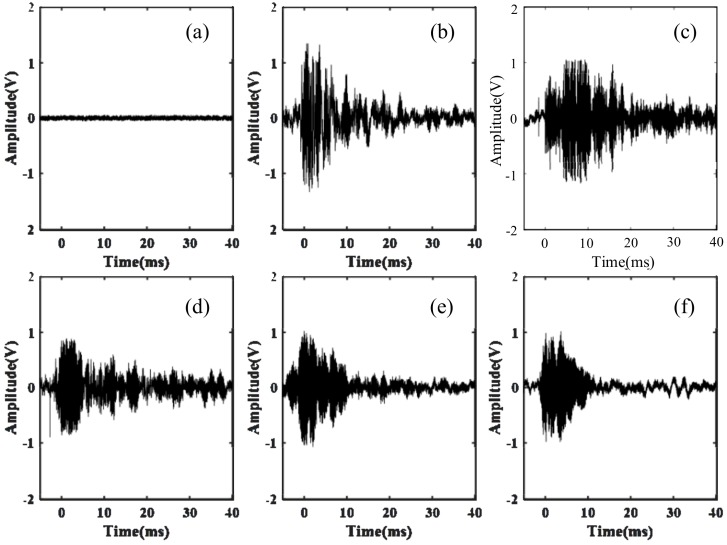
Time-domain waveform of PD signals at different positions. (**a**) No partial discharge event. The sensing probe is placed (**b**) on the top, (**c**) in the front, (**d**) behind, (**e**) on the left side and (**f**) on the right side of the discharge protection hood.

Figure 10 shows the time-domain waveform of PD signals collected by the optical fiber sensing probe with 40 laps. Port 3 of coupler B was connected with balance detector E and the data was collected by the DAQ card. Overall, it could be seen that the waveform attenuation was similar and the amplitude of the PD signals ranged approximately between 0.8 V–1.2 V in the time-domain graph according to the different locations. In no PD event was the amplitude of the PD signals 0 V. When there were PD signals, the waveform of the PD signals detected by the optical fiber sensing probe in different positions had obvious pulse signals and the waveform was similar. The positive and negative amplitudes of the PD signals had obvious asymmetry. When the optical fiber sensing probe was located on the top, the amplitude of the PD signals was the most obvious and up to 1.2 V. To further explore the influence of different optical fiber sensing lap numbers on PD signals, the optical fiber sensing probe was placed on the top of PD model to carry out the experiments below.

**Figure 11:**
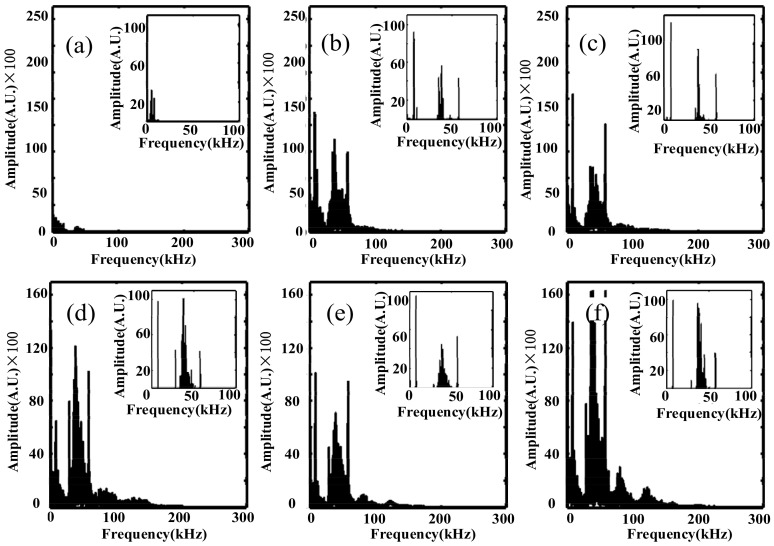
Frequency waveform of PD signals at different positions. (**a**) No partial discharge event. The sensing probe was placed (**b**) on the top, (**c**) in the front, (**d**) behind, (**e**) on the left side and (**f**) on the right side of the discharge protection hood. The zoomed figures in every sub-figure show the frequency distribution histogram.

Figure 11 shows the frequency waveform of the PD signals collected by the optical fiber sensing probe with 40 laps in different positions. The frequency distribution curve of the PD signals was obtained by Fourier transform of the time-domain signal collected by the experiment. In general, it could be seen that the frequency range was about 10 kHz–60 kHz. In no PD event was the frequency of the PD signals less than 8 kHz, and the amplitude of frequency was very small, which was the frequency of the environmental noise. When there were PD signals, the frequency waveform of the PD signals detected from the different positions of the optical fiber sensing probe had obvious frequency distributions. The waveform was basically similar. When the optical fiber sensing probe was located on the top, the amplitude of the frequency was the most obvious.

In order to analyze the frequency of the PD signals, the 110 sets of data were collected by the optical fiber sensing probe in different directions and the spectrum of PD signals was obtained using the Fourier transform method. According to the maximum value of the spectrum of the PD signals in different positions, the highest amplitude was multiplied by 0.5 as the threshold. The data above the threshold value were then accumulated, and we retrieved the frequency distribution histogram as shown in Figure 7 through the zoomed figures in every sub-figure.

**Figure 12:**
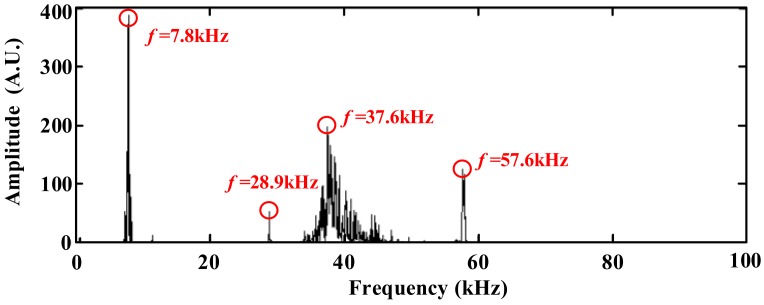
Characteristic frequency distribution diagram using OFPDS.

Based on the above experiments, we retrieved the time and frequency domain waveform of the PD signals. In order to verify the characteristic frequency of the PD signals in the frequency domain, Figure 12 showed the frequency distribution histograms of the OFPDS. In Figure 12, this frequency distribution between 28.9 kHz and 57.6 kHz was calculated by the accumulation of all the frequency distribution histograms that had been obtained in six positions by the OFPDS. The characteristic frequency distribution diagram of the PDs using OFPDS was distributed at 7.8 kHz, 28.9 kHz, 37.6 kHz and 57.6 kHz. By using the cumulative histogram method, the characteristic ultrasonic frequency band of PD was between 28.9 kHz and 57.6 kHz for this OFPDS.

### 4.3. Influence of the Sensing Head

**Figure 13:**
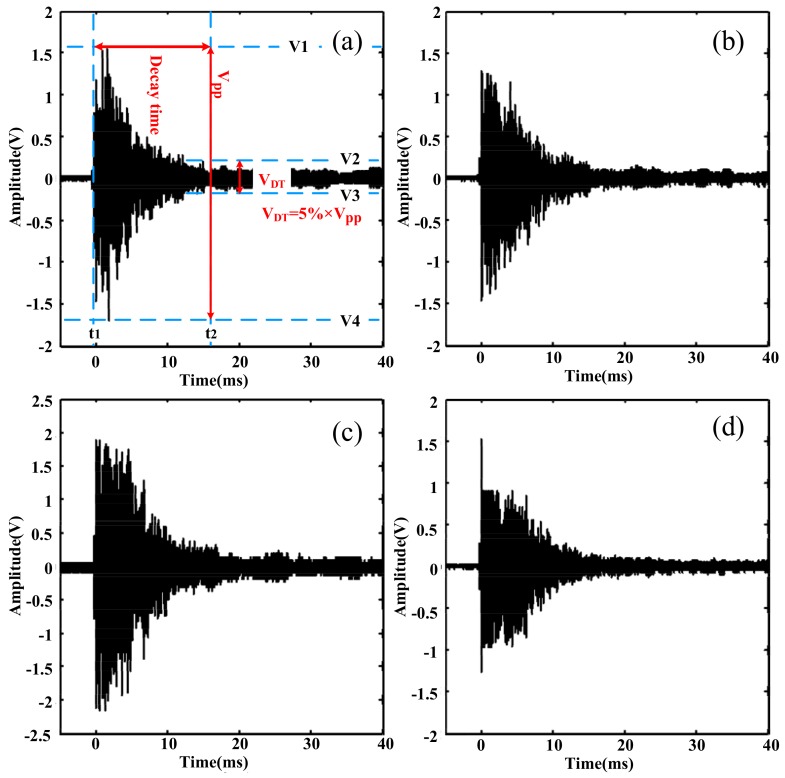
The time domain waveform of the detected PD signals with (**a**) 10-lap fiber probe, (**b**) 40-lap fiber probe, (**c**) 70-lap fiber probe and (**d**) 100-lap fiber probe.

In order to improve the sensibility of the fiber-optic method and obtain more typical frequency components besides 28.9 kHz and 58 kHz, the influence of the optical fiber sensing probe on PD detection was preliminarily investigated. Here, the optical fiber probe was placed on the top of the discharge protection hood because of its higher signal-to-noise ratio, shown in Figure 13. Figure 13 shows the different time-domain PD signals detected through the OFPDS by means of the variation of the lap number of the optical fiber sensing probe. Three variables—V_pp_, V_DT_, and decay time—were defined. V_pp_ was the maximum peak–peak value, the instant of which is t_1_, and V_DT_ was equal to 5% of V_PP_, the instant of which is t_2_. The decay time was defined as the difference between t_2_ and t_1_, as shown in Figure 13a. On the whole, it was clear that the V_pp_ of the PD signals ranged approximately between 0.9–2 V in the time-domain graph for the cases with different laps of the optical fiber sensing probe.

To analyze the time-domain characteristics of the PD signals, several experiments were carried out in the same laboratory environment by changing the lap number of the optical fiber sensing probe, which was realized at a length of 4.1 km from the output of the 2 × 1 coupler. The sensing probe was always placed on the top of the PD simulator device. We also made the needle–needle discharge in an orderly manner by triggering the switch button. The lap number of the optical fiber sensing probe increased with a step of 10 laps, from 10 laps to 100 laps.

**Figure 14:**
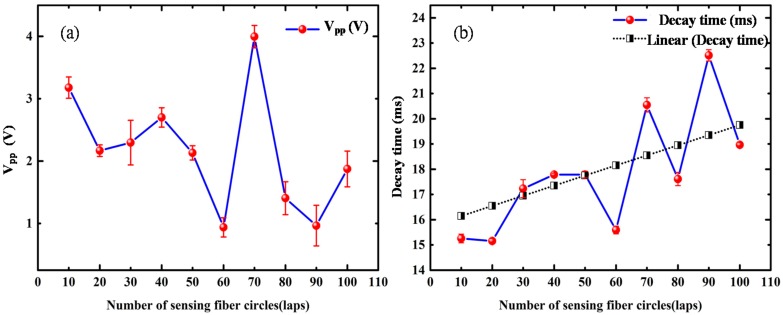
The variations of (**a**) V_pp_ and (**b**) decay time versus the number of sensing fiber circles.

Figure 14 shows the variation of V_pp_ and decay time with the number of sensing fiber circles. Figure 14a shows that the overall trend of V_pp_ was nonlinear and fluctuant within 100 laps; this means that the number of sensing fiber circles was not the only influencing factor for the sensing performance in the time-domain. The large fluctuations with some laps reflect that other factors such as fiber looseness may have played a role. Figure 14b shows that as the number of sensing fiber circles increased, the decay time was strongly influenced by the lap number, with a trend of fluctuant growth. There was always an exponential decay and oscillation in the detected excitation ultrasonic signal. With the increase in the number of sensing fiber circles, the sensing system could detect more decay components, because the affecting time upon the phase shift in the optical fiber caused by the propagating ultrasonic signal also increased.

**Figure 15:**
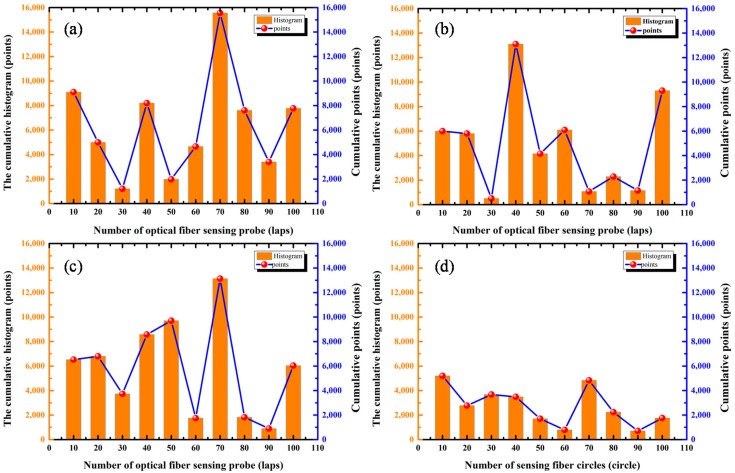
Statistical histogram of the frequency of (**a**) 7.8 kHz, (**b**) 28.9 kHz, (**c**) 37.6 kHz and (**d**) 57.6 kHz with the number of sensing fiber circles.

In addition, we investigated the influence of the optical fiber sensing probe on ultrasonic signals of PD from the frequency domain waveform. Figure 12 shows that there were obvious characteristic frequency components at 7.8 kHz, 28.9 kHz, 37.6 kHz and 57.6 kHz. Therefore, these frequency components were analyzed statistically, and the results are shown in the following Figure 15.

Figure 15 shows that the cumulative histogram and the cumulative points of the frequency intensity of the detected ultrasonic signals varied with the number of sensing fiber circles. With a certain lap number, the cumulative histogram and points had the maximum value, for example, 70 laps for 7.8 kHz (as shown in Figure 15a) and 37.6 kHz (as shown in Figure 15c), 40 laps for 28.9 kHz (as shown in Figure 15b), and 10 laps for 57.6 kHz (as shown in Figure 15d). In addition, the overall frequency response of 57.6 kHz was smaller than other frequency components. From the above analysis, we can see that there was an obvious frequency selectivity in the PD ultrasonic signal, even if the sensing probe was changed with the number of sensing fiber circles. This means that these characteristic frequency components could be used for the detection evaluation of the PD ultrasonic signal.

**Figure 16:**
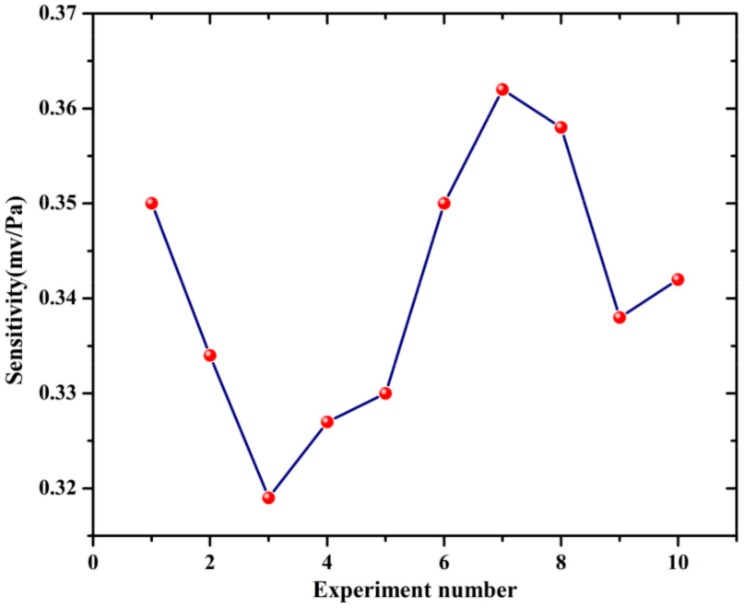
The sensitivity of optical fiber PD sensing system.

Figure 16 shows the sensitivity of the optical fiber PD sensing system. A total of 10 groups of repetitive experiments were carried out, in which the distance between the sound pressure meter and the ultrasonic transducer was 1 cm, the excitation ultrasonic signal was 25 kHz with 20 V. The detected V_pp_ by the optical fiber sensing probe was about 7 mV and the average sound pressure level was 120.23 dB. Thus, the sensitivity of this system was able to be obtained according to the following equation:

(16) $$S=20\mathrm{lg}\frac{X\mathrm{Pa}}{20\times 10^{-6}\mathrm{Pa}}\mathrm{dB}$$

where *S* was sound pressure level, *X* was the sound pressure to be measured, the reference sound pressure in air was 20 × 10^−6^ Pa. Thus, the detected sound pressure was about 20.54 Pa, and the sensitivity of the optical fiber PD sensing system varied between 0.32 mV/Pa and 0.36 mV/Pa. As the amplitude of vibration was usually weaker when the frequency was higher, particular in the ultrasound range, the amelioration of the sensitivity was a challenging task; this needs to be considered with regard to other novel propositions for optical methods.

## 5. Conclusions 

In this paper, the characteristics of PD were analyzed, an optical fiber sensing probe structure for detecting the ultrasonic signals of PD was designed, and a PD detection scheme based on linear Sagnac optical interferometry was proposed. The standard ultrasound signal was used to verify the feasibility of the optical fiber method. In addition, the frequency range of the OFPDS was verified and the time domain and the frequency of PD using the optical fiber method was analyzed. Experiments showed that with 10 kV voltage, the time-domain amplitude range of the PD signals was 0.8~1.9 V, and the frequency response range was up to 58 kHz. This method provided a new scheme for the PD detection of power equipment, such as transformers, high voltage switchgear, power cables and so on. In the next step, we will study the PD detection method of linear Sagnac optical interferometry and focus on the identification of different types of PD mode fiber-optic recognition.
